# *Toxoplasma* Uses GRA16 To Upregulate Host c-Myc

**DOI:** 10.1128/mSphere.00402-20

**Published:** 2020-06-24

**Authors:** Michael W. Panas, John C. Boothroyd

**Affiliations:** a Department of Microbiology and Immunology, Stanford School of Medicine, Stanford, California, USA; University of California, Davis

**Keywords:** GRA16, c-Myc, effector protein, host-microbial interaction

## Abstract

Manipulation of the host cell is a crucial part of life for many intracellular organisms. We have recently come to appreciate the extent to which the intracellular pathogen Toxoplasma gondii reprograms its host cell, and this is illustrated by the marked upregulation of the central regulator c-Myc, an oncogene that coordinates myriad cellular functions. In an effort to identify an effector protein capable of regulating c-Myc, our laboratory constructed a screen for mutant parasites unable to accomplish this upregulation. Interestingly, this screen identified numerous components of a complex located in/on the parasitophorous vacuole membrane necessary to translocate *Toxoplasma* proteins out into the host cytosol, but it never identified a specific effector protein. Thus, how the parasite upregulates c-Myc has largely been a mystery. Previously, the *Toxoplasma* dense granule protein GRA16 has been described to bind to one isoform of PP2A-B, a regulatory subunit that coordinates the activity of the catalytic protein phosphatase PP2A. As other PP2A subunits have been reported to target PP2A protein phosphatase activity to c-Myc, subsequently leading to c-Myc destabilization, we examined whether GRA16 has an impact on host c-Myc accumulation. Expression of *Toxoplasma*’s GRA16 protein in Neospora caninum, a close relative of *Toxoplasma* that does not naturally upregulate host c-Myc, conferred the ability on *Neospora* to do this now. Further support was obtained by deleting the *GRA16* gene from *Toxoplasma* and observing a severely diminished ability of *Toxoplasma* tachyzoites to upregulate host c-Myc. Thus, GRA16 is an effector protein central to *Toxoplasma*’s ability to upregulate host c-Myc.

**IMPORTANCE** The proto-oncogene c-*Myc* plays a crucial role in the growth and division of many animal cells. Previous studies have identified an active upregulation of c-Myc by *Toxoplasma* tachyzoites, suggesting the existence of one or more exported “effector” proteins. The identity of such an effector, however, has not previously been known. Here, we show that a previously known secreted protein, GRA16, plays a crucial role in c-Myc upregulation. This finding will enable further dissection of the precise mechanism and role of c-Myc upregulation in *Toxoplasma*-infected cells.

## Abstract (precis)

The proto-oncogene c-*Myc* plays a crucial role in the growth and division of many animal cells. Previous studies have identified an active upregulation of c-Myc by *Toxoplasma* tachyzoites, suggesting the existence of one or more exported “effector” proteins. The identity of such an effector, however, has not previously been known. Here, we show that a previously known secreted protein, GRA16, plays a crucial role in c-Myc upregulation. This finding will enable further dissection of the precise mechanism and role of c-Myc upregulation in *Toxoplasma*-infected cells.

## OBSERVATION

The ability of a parasite to control its host’s gene expression potentially allows that parasite to remodel the host, prevent apoptosis, access nutrients, and blunt specific and nonspecific immune functions. During the intracellular growth phase of Toxoplasma gondii, dense granule proteins are secreted into the parasitophorous vacuole. Some of these proteins are then translocated across the parasitophorous vacuole membrane (PVM) into the host cytosol, in some cases ultimately reaching the host nucleus where they can impact transcription.

In total, more than 2,000 host genes are impacted by GRA effector proteins (1). The broad impact of such effectors is no doubt due in part to the fact that they target critical regulators of numerous pathways, such as cell growth, cytokine production, cholesterol homeostasis, and beta-catenin signaling (2–5). One crucial linchpin of gene expression that the parasite targets is c-Myc, a central regulator capable of controlling more than 15% of the genes in the mammalian genome (6). Previous genetic screens showed that upregulation of c-Myc is dependent on a number of *Toxoplasma* genes, including four dubbed “Myc regulatory” genes (MYRs) (7). In all cases, however, these MYR genes were shown to be necessary for the translocation of many GRA effectors across the PVM, rather than encoding the protein directly controlling c-Myc upregulation. This indicated that c-Myc expression is modulated by a MYR-dependent effector, but the identity of that effector has not previously been determined.

The ability to upregulate human c-Myc is a trait not shared by *Toxoplasma*’s close relative, Neospora caninum (8), even though *Neospora* contains orthologues of the MYR system as well as orthologues of several dense granule proteins, including GRA16 (5). *Neospora*’s inability to upregulate human c-Myc could arise from a complete lack of the c-Myc-regulating protein(s) or evolutionary adaptation of those proteins in a way that causes them to be specifically unable to affect human c-Myc expression.

The accumulation of c-Myc is dependent upon its stabilization; without phosphorylations on key conserved residues, c-Myc is actively degraded. Part of the machinery regulating the stability of c-Myc is the PP2A holoenzyme, a complex that can dephosphorylate the Ser62 residue on c-Myc. PP2A consists of a catalytic core, a structural alpha subunit, and one of numerous beta-targeting subunits. Specifically, the PP2A complex dephosphorylates c-Myc when the PP2A-C catalytic component is complexed to beta subunit PP2AB56α (B56α), leading to c-Myc degradation (9). Conversely, when PP2A cannot complex with the B56α subunit and target c-Myc, c-Myc remains phosphorylated at serine 52, and this leads to overaccumulation (10, 11). The C-terminal portion of GRA16 was shown to bind to the PP2AB55 (B55) subunit, pulling down the entire PP2A complex (5). While an association with the B56α subunit that catalyzes c-Myc instability was not reported with GRA16, we reasoned that GRA16-stabilized complexes containing the B55 isoform may interfere in the accumulation of c-Myc and therefore sought to test whether GRA16 plays a role in the upregulation of human c-Myc.

## 

### Experimental findings.

**FIG 1:**
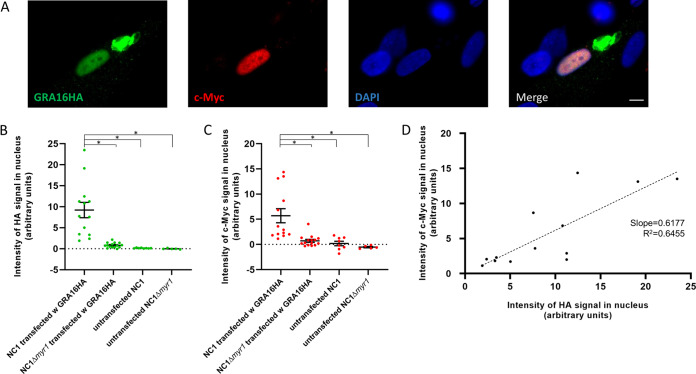
*Neospora* transfected with *TgGRA16HA* induces host c-Myc. (A) Representative image of human foreskin fibroblasts (HFFs) infected for 18 h with Neospora caninum NC1 transiently transfected with a plasmid carrying the gene coding for *Toxoplasma*’s GRA16 with an HA tag. Staining for HA (green), staining for host c-Myc (red), and DAPI staining of all nuclei (blue) were performed. Bar, 5 μm. (B) Quantitation of the intensity of HA in the nuclei of cells infected with the indicated strains. Data are representative of two independent biological replicates, with between 5 and 15 infected cells analyzed in each. Asterisks indicate significant (*P* < 0.01) differences between the indicated conditions. (C) As for panel B except the intensity of nuclear c-Myc staining was quantified. (D) Correlation between the level of GRA16HA in the nucleus of each cell infected with NC1 transfected with GRA16HA and the level of c-Myc expression within that same nucleus.

To test the ability of *Toxoplasma* GRA16 to impact host c-Myc expression, we transiently transfected a plasmid containing the *GRA16* gene with a hemagglutinin (HA) tag into a wild-type population of Neospora caninum strain NC1, which was then allowed to infect human foreskin fibroblast (HFF) cells. About 18 h later, an immunofluorescence assay (IFA) was used to identify host cells infected with *Neospora* expressing the GRA16HA, and simultaneously, we assessed the level of nuclear c-Myc expression in those cells. Cells harboring untransfected NC1 parasites served as negative controls. The results showed, first, that GRA16HA expressed in *Neospora* is exported to the parasitophorous vacuole from where it ultimately reaches the host nucleus, albeit to various degrees (Fig. 1A and B). To test whether this process is dependent on MYR1, as it is in *Toxoplasma* (12), we repeated these experiments in an NC1 strain harboring a disruption of the *MYR1* orthologue (NC1Δ*myr1*). The results (Fig. 1B) showed that the NC1Δ*myr1* strain does not efficiently translocate GRA16HA across the PVM. Together, these results show that *Neospora* has the machinery necessary for translocation of a *Toxoplasma* GRA effector across the PVM and that, as for *Toxoplasma*, this machinery is dependent on the *MYR1* gene. The second observation we made in these experiments was that expression of GRA16 in *Neospora* was associated with a variable but marked increase in the expression of c-Myc in the infected host cell’s nucleus (Fig. 1A and C). Moreover, we observed a positive correlation between the intensities of GRA16HA and c-Myc staining in any given nucleus (Fig. 1D), and analysis using an F test demonstrates that this difference is significantly different from a slope of zero (*P* = 0.0009). These results indicate that *Toxoplasma* GRA16 plays a pivotal role in the induction of host c-Myc in infected cells.

**FIG 2:**
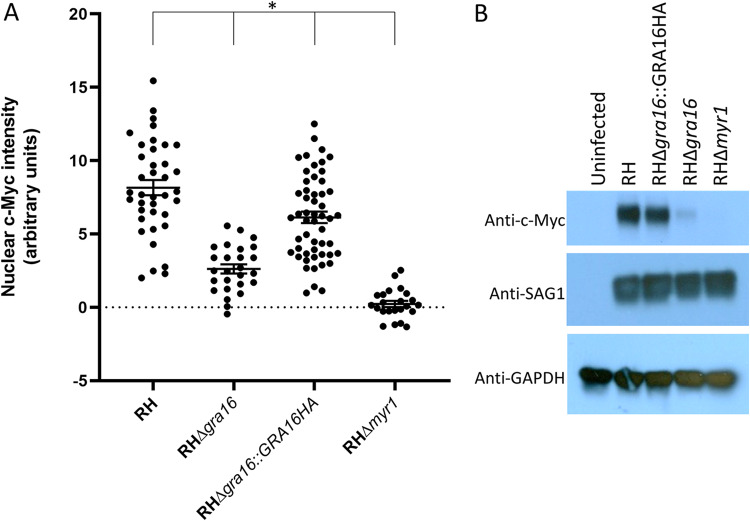
Deletion of *GRA16* in *Toxoplasma* reduces the upregulation of host c-Myc expression. (A) c-Myc expression in HFFs infected with the indicated strain for 18 h was assessed by IFA and quantified by ImageJ analysis. Data represent one of two biological replicates that yielded essentially similar results. For RH, *n* = 38; for RHΔ*gra16*, *n* = 27; for RHΔ*gra16*::*GRA16HA*, *n* = 53; for RHΔ*myr1*, *n* = 23. The asterisk indicates that all groups are statistically different from each other (*P* < 0.01). (B) Western blot analysis of c-Myc expression from HFFs infected for 18 h with the same strains as in panel A. The same blot was stripped and reprobed with anti-SAG1 antibody to assess parasite burden and glyceraldehyde-3-phosphate dehydrogenase (GAPDH) to assess gel loading. Images represent one of three blots performed, all showing similar results.

A prediction of these results is that deletion of *GRA16* from *Toxoplasma* should substantially reduce their ability to upregulate host c-Myc. To test this prediction, we used CRISPR-Cas9 to engineer disruptions at the 5′ end of the single exon encoding GRA16 in the *Toxoplasma* RH strain. A complementation control was also generated by integrating a plasmid encoding GRA16HA (13) into the *UPRT* locus of the RH*Δgra16* strain and then selecting with 5-fluorodeoxyuridine (FUDR). The ability of these strains to upregulate c-Myc was then assessed by IFA and compared to infection with positive (wild-type RH) and negative (RHΔ*myr1*) control strains 18 h postinfection. The results (Fig. 2A) showed that infection with the RH*Δgra16* strain resulted in substantially lower host c-Myc induction compared to the wild-type RH strain, although the magnitude of the decrease was less than that seen for the RHΔ*myr1* strain. Complementation of the RHΔ*gra16* strain with a wild-type copy of *GRA16* recovered c-Myc induction to nearly the levels seen with the wild-type RH strain, although there remained a statistically significant difference between all four groups. To confirm this result, samples were also harvested for Western blot analysis following 18 h of infection with the same four strains of *Toxoplasma*. Lysates of these samples were resolved by gel electrophoresis, blotted, and stained with the Y69 antibody recognizing c-Myc; the results (Fig. 2B) showed that RH and the complemented RHΔ*gra16*::GRA16HA strain induce robust c-Myc expression, while the GRA16 deletion eliminates almost all of this upregulation. In cells infected with parasites lacking MYR1, no c-Myc expression was observed.

### Discussion.

The results reported here show that *Toxoplasma* GRA16 plays a crucial role in the accumulation of host c-Myc. How *Toxoplasma* GRA16 drives this upregulation is still not clear, although its interaction with PP2A and the B55 subunit does provide an attractive hypothesis: by binding to and stabilizing the PP2A complex with B55 subunits, specifically excluding the B56α subunits that normally target c-Myc for phosphorylation, the parasite might be able to prevent degradation of c-Myc.

Our results suggest that GRA16 is not the only mechanism through which *Toxoplasma* impacts host c-Myc levels, as deletion of *GRA16* eliminated only about 68% of the response, whereas deletion of *MYR1* caused essentially a complete ablation (97% reduction). Thus, GRA16 might operate in conjunction with another MYR1-dependent effector. Such a hypothesis would explain why no effector proteins were identified in the genetic screen for mutants unable to upregulate c-Myc expression (12); that screen was done after only a mild mutagenesis and mutants defective in just one effector might yield a partial defect that a stringent selection would not pull out, whereas a defect in the effector translocation machinery (e.g., the MYR proteins) would block the export of all effectors and produce a completely null phenotype. Our data, combined with that of Bougdour et al. (5), suggest this unidentified second effector might work through a complementary mechanism, such as upregulating expression of c-Myc mRNA. Possibilities for the identity of such an effector include GRA24, as its target, p38 mitogen-activated protein (MAP) kinase, can impact cell cycle progression (14), and/or HCE1/TEEGR as it upregulates expression of cyclin E and CDK2 (3), a complex that can indirectly as well as directly phosphorylate c-Myc (15).

Our results also confirm previous work (3) showing that *Neospora* can express and translocate a *Toxoplasma* dense granule protein across the PVM. Here, we extend this finding by showing that this translocation by *Neospora* requires its MYR1 orthologue, arguing for a common means for recognizing effectors to be exported between the MYR machinery in these two closely related parasites. Interestingly, *Neospora* carries a gene that encodes a *GRA16* homologue, although no function has yet been reported for it; this work supports the conclusion, however, that *Neospora* is unable to upregulate host c-Myc because of divergence in its effector protein, not because of the remote but still conceivable possibility that it lacks a functional export machinery. The divergence between the *Neospora* and *Toxoplasma* orthologues of GRA16 is most striking in the C-terminal region, where *Neospora* GRA16 lacks 21 amino acids present in *Toxoplasma* GRA16. As the C-terminal domain has previously been shown to be necessary for *Toxoplasma* GRA16 binding to PP2A B55, it is possible that this difference explains *Neospora* GRA16’s inability to activate c-Myc. We note, however, that these two GRA16 orthologues are markedly different throughout their entire length (only 37% identity, overall) so other regions might also be involved.

Last, it seems likely that *Toxoplasma*’s upregulation of c-Myc is a selected trait, rather than incidental, as other parasites appear to have also evolved a similar ability. In the case of *Theileria*, for example, c-Myc upregulation provides an antiapoptotic effect that lasts for the duration of the parasite infection, but not after (16); likewise, a genetic screen for host factors necessary for *Microsporidia* development suggests that the Myc family of transcription factors promote pathogen development (17). In the case of *Toxoplasma*, the impact or benefit of c-Myc upregulation is not known, but it could play a role in the reported alteration of the host cell cycle (18–20), although if so, GRA16 would be one of a few parasite factors engaged in this crucial process; i.e., GRA16 would be operating in conjunction with GRA24 and/or ROP38, both of which impact p38 MAP kinase (2, 21), and HCE1/TEEGR which alters cyclin E expression (3). Further work will be needed to dissect the precise mechanisms by which GRA16 operates as well as the impacts of c-Myc upregulation on *Toxoplasma*’s interaction with its hosts.

### Experimental procedures.

**(i) Strains used.** Toxoplasma gondii RHΔ*hpt*, Neospora caninum NC1, and specified derivatives of these strains were used for this study. Tachyzoites were maintained by serial passage in human foreskin fibroblasts (HFFs) cultured in complete Dulbecco’s modified Eagle medium (cDMEM) supplemented with 10% heat-inactivated fetal bovine serum (FBS), 2 mM l-glutamine, 100 U/ml penicillin, and 100 μg/ml streptomycin and grown at 37°C in 5% CO_2_. Infections included in this study were performed by scraping infected monolayers and lysing the host cells open using a 27-gauge needle. The released parasites were counted using a hemocytometer and added to confluent HFFs at the multiplicity of infection (MOI) stated.

**(ii) Immunofluorescence microscopy.** Immunofluorescence assays were performed as previously described (22). Briefly, infected cells grown on glass coverslips were fixed using 4% formaldehyde at room temperature (RT) for 20 min, washed with phosphate-buffered saline (PBS), permeabilized with 0.2% Triton X-100 for 20 min, and blocked with PBS with 3% bovine serum albumin (PBS-3% BSA). GRA16HA was detected using rat anti-HA antibodies (3F10; Roche), while host c-Myc was detected using monoclonal antibody Y69 (AB32072; Abcam). Primary antibodies were detected with goat polyclonal Alexa Fluor-conjugated secondary antibodies (Invitrogen). Vectashield with 4′,6′-diamidino-2-phenylindole (DAPI) stain (Vector Laboratories) was used to mount the coverslips on slides. Fluorescence was detected using an epifluorescence microscope.

**(iii) Quantitation of nuclear GRA16HA and c-Myc.** Quantitation of nuclear intensity was performed as previously described (22). Briefly, phase-contrast, DAPI, anti-HA, and anti-c-MYC images were taken of 10 to 20 fields of view and analyzed using ImageJ. All images shown for any given condition/staining in any given comparison/data set were obtained using identical parameters. To assess the amount of GRA16HA or c-Myc in a nucleus, phase contrast was used to define the infected cells of these images, then ImageJ was used to define the nucleus on the DAPI-stained corresponding images, and these nuclear boundaries were then quantified using the ImageJ “measure” for the intensity of GRA16HA intensity and on the corresponding c-Myc stained images.

**(iv) Gene disruption and complementation.** Genetic manipulation of the GRA16 locus was performed as previously described (22). The RHΔ*gra16* strain was generated by disrupting the corresponding gene locus using CRISPR-Cas9 and selecting for integration of a linearized vector encoding hypoxanthine-guanine phosphoribosyl transferase (HXGPRT) using drug selection for 8 days with 25 μg/ml mycophenolic acid (MPA) and 50 μg/ml xanthine (XAN) for HXGPRT selection. Specifically, the pSAG1:U6-Cas9:sgUPRT vector (23) was modified by Q5 site-directed mutagenesis (NEB) using primer F16 (GCCTGCAGGCTCTTCGAAGTGTTTTAGAGCTAGAAATAGC) to specify a single guide RNA (sgRNA) targeting *TGGT1_208830*. The resulting sgRNA plasmid, dubbed pSAG1:U6-Cas9:sgGRA16 was transfected into the RHΔ*hpt* strain of *Toxoplasma* with 3 μg pTKO2 (HXGPRT+) plasmid. The parasites were allowed to infect HFFs in 24-well plates for 24 h, after which the medium was changed to complete DMEM supplemented with 50 μg/ml MPA and 50 μg/ml XAN for HXGPRT selection, passed twice, and then single cloned into 96-well plates by limiting dilution. Disruption of the gene coding regions was confirmed by PCR and sequencing of the locus.

**(v) Creation of complemented lines.** The RHΔ*gra16* strain was complemented ectopically by mixing parasites in P3 buffer with the sgUPRT plasmid (24) and pGRA16-HA plasmid (13) and then electroporating on the Amaxa 4D Nucleofector (Lonza). Selection with 5 μM FUDR for 1 week was then followed by single cloning by limiting dilution and testing for HA expression by IFA.

**(vi) Western blotting.** Western blotting was performed as previously described (12).

**(vii) Statistical analyses.** Statistical analysis was performed with Prism version 8.0.2 software. An F test was used to determine whether the slope of regression lines significantly differed from zero. Differences in intensity were analyzed by one-way analysis of variance (ANOVA) with a *posthoc* Tukey’s test.
